# Design of a Randomized Controlled Clinical Study of tissue-engineered osteogenic materials using bone marrow-derived mesenchymal cells for Maxillomandibular bone defects in Japan: the TEOM study protocol

**DOI:** 10.1186/s12903-019-0753-1

**Published:** 2019-04-30

**Authors:** Shinobu Shimizu, Shuhei Tsuchiya, Akihiro Hirakawa, Katsuyoshi Kato, Masahiko Ando, Masaaki Mizuno, Masashi Osugi, Kazuto Okabe, Wataru Katagiri, Hideharu Hibi

**Affiliations:** 1 Center for Advanced Medicine and Clinical Research, Nagoya University Hospital, 65 Tsurumai-cho, Showa-ku, Nagoya, Aichi 466-8560 Japan; 2 Department of Oral and Maxillofacial Surgery, Nagoya University Hospital, 65 Tsurumai-cho, Showa-ku, Nagoya, Aichi 466-8560 Japan; 3 Department of Biostatistics and Bioinformatics, Graduate School of Medicine, The University of Tokyo, 7-3-1 Hongo, Bunkyo-ku, Tokyo, 113-0033 Japan; 4 Department of Oral and Maxillofacial Surgery, Fujieda Heisei Memorial Hospital, 123-1 Mizukami, Fujieda, Shizuoka, 426-8662 Japan; 5 Division of Reconstructive Surgery and Oral and Maxillofacial Region, Niigata University Graduate School of Medical and Dental Sciences, 2-5274 Gakkocho-dori, Chuo-ku, Niigata, 951-8514 Japan; 6 Department of Oral and Maxillofacial Surgery, Nagoya University Graduate School of Medicine, 65 Tsurumai-cho, Showa-ku, Nagoya, Aichi 466-8550 Japan

**Keywords:** Tissue engineering, Regenerative medicines, Cell therapy, Bone marrow cells, Cell transplantation, Bone, Clinical study

## Abstract

**Background:**

Maxillomandibular bone defects arise from maxillofacial injury or tumor/cyst removal. While the standard therapy for bone regeneration is transplantation with autologous bone or artificial bone, these therapies are still unsatisfactory. Autologous bone harvesting is invasive and occasionally absorbed at the implanted site. The artificial bone takes a long time to ossify and it often gets infected. Therefore, we have focused on regenerative therapy consisting of autologous bone marrow-derived mesenchymal cells (BM-MSCs), which decreases the burden on patients. Based on our previous research in patients with maxillomandibular bone defects or alveolar bone atrophy using a mixture of BM-MSCs, platelet-rich plasma (PRP), thrombin, and calcium, we confirmed the efficacy and acceptable safety profile of this treatment. In this investigator-initiated clinical study (the TEOM study), we intended to add β-tricalcium phosphate (β-TCP) owing to large defect with patients. The TEOM study aimed to evaluate the efficacy and safety of bone regeneration using mixtures of BM-MSCs in patients with bone defects resulting from maxillofacial injury, and tumor/cyst removal in the maxillomandibular region.

**Methods:**

The TEOM study is an open-label, single-center, randomized controlled study involving a total of 83 segments by the Fédération Dentaire Internationale numbering system in maxillomandibular bone defects that comprise over 1/3 of the maxillomandibular area with a remaining bone height of ≤10 mm. The primary endpoint is rate of procedure sites with successful bone regeneration defined as a computed tomography (CT) value of more than 400 and a bone height of more than 10 mm. Our specific hypothesis is that the number of required regions was calculated assuming that the rate of procedure sites with successful bone regeneration is similar and the non-inferiority margin is 15.0%.

**Discussion:**

The TEOM study is the first randomized controlled study of regenerative treatment using BM-MSCs for large maxillomandibular bone defects. We will evaluate the efficacy and safety in this study to provide an exploratory basis for the necessity of BM-MSCs for these patients.

**Trial registration:**

This trial was registered at the University Hospital Medical information Network Clinical Trials Registry (UMIN-CTR Unique ID: UMIN000020398; Registration Date: Jan 15, 2016; URL: https://upload.umin.ac.jp/cgi-open-bin/ctr_e/ctr_view.cgi?recptno=R000016543).

## Background

Maxillomandibular bone defects occur as a result of maxillofacial injury or surgery for maxillofacial tumors/cysts. Individuals with maxillomandibular bone defects may face limits in their daily living activities because of difficulty of eating without teeth, and unusual facial features; hence, it can have a major impact on the quality of life (QOL) of patients. While the standard therapy for these conditions is transplantation of autologous bone from the ilium, the tibia and any other bones, it is invasive for patients and hospitalization is necessary, further side effects such as infection and hematoma can arise [1]. In addition, bone grafts are occasionally absorbed at the implanted site depending on time [2, 3]. The other currently used therapy is the application of artificial bone (e.g. β-TCP, hydroxyapatite) at the defect area [4]; however, it is known that it takes a long time for ossification in cases of large defects and the implanted site often gets infected [5]. Another potential therapy is using a growth factor such as BMP-2 [6], but its efficacy has not been established in Japan and severe complications (e.g. edema in head and neck region) arise occasionally [7]. These treatments are still unsatisfactory; therefore, new regenerative therapies are required.

Meanwhile, bone regenerative cell therapy has been focused on in several experimental studies [8, 9]. We previously confirmed that transplantation of cultured BM-MSCs with PRP, thrombin, and calcium improved bone volume in non-clinical and clinical studies in patients with maxillomandibular bone defects or alveolar bone atrophy [10–19]; however, these clinical studies were a non-comparative single-arm design. Therefore, we planned a preliminary randomized controlled clinical study with the objective of confirming the efficacy of BM-MSCs in patients with maxillomandibular bone defects. In other words, we compare the efficacy and safety clinically of the mixture of “PRP, thrombin, calcium, β-TCP, and BM-MSCs” (BM-MSCs group), or the mixture of “PRP, thrombin, calcium, and β-TCP” (control group). We intend to add β-TCP as a prosthetic bone due to patients with larger defects in this clinical study than in previous clinical studies [10, 13–19].

In this article, we provide the detailed design of this investigator-initiated clinical study in adult jawbone defect patients as an exploratory study in Japan (the TEOM study). Incidentally, the main design of this trial is undergoing the process of approval by the Advanced Medical Care B program of the Ministry of Health, Labour and Welfare (MHLW), which is one of the unique systems in Japan and able to evaluate as a novel medical technology whether to be approved by the Central Medical Council on Social Insurance for coverage by public health insurance or not, based on its efficacy and safety [20].

## Methods/design

### Overall design and objective

**Fig. 1:**
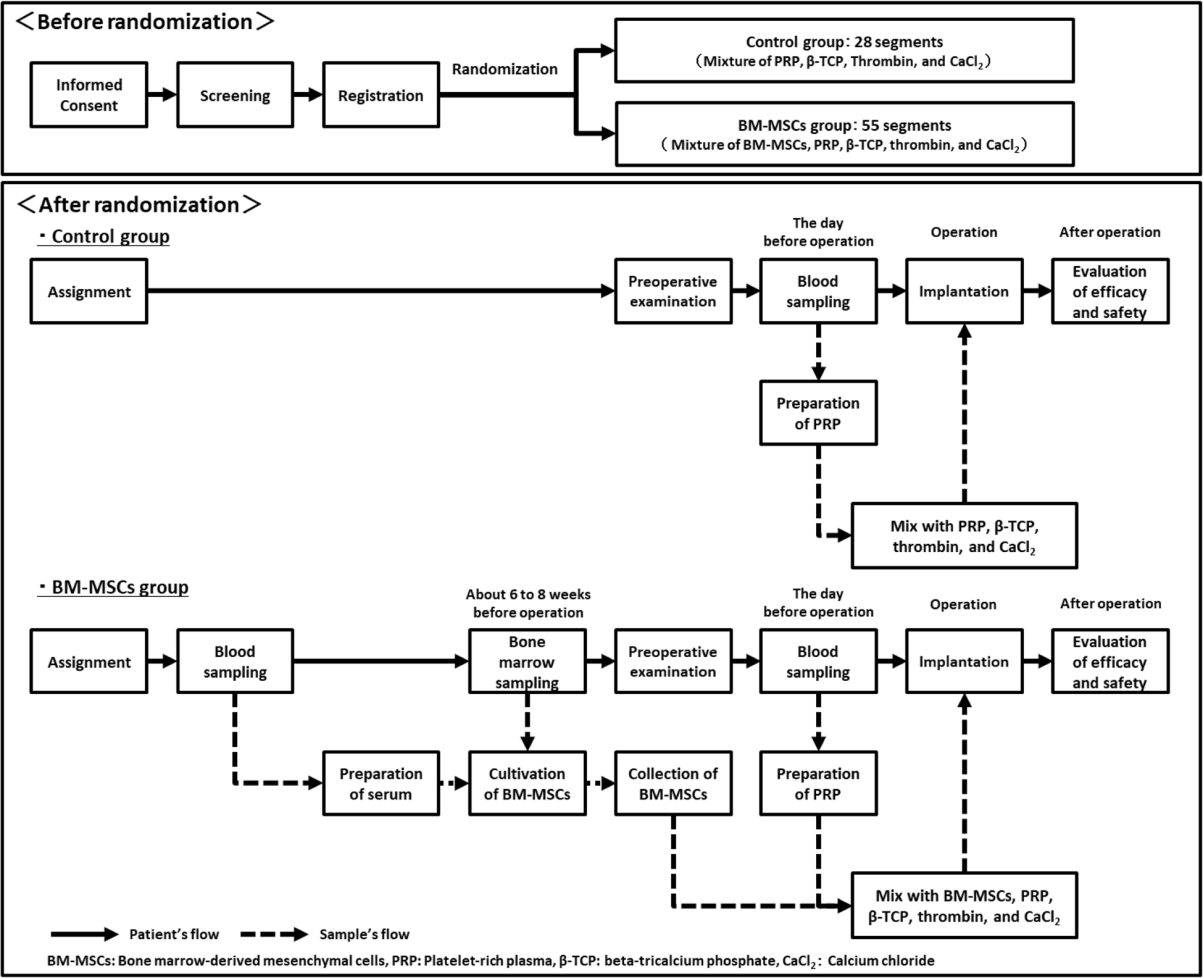
Flow diagram of the TEOM study. This flow diagram is consisted of 2 step, ‘Before randomization’ and ‘After randomization’. In the ‘Before randomization’, patients will be randomized to BM-MSCs group or control group. The outline of procedure in each group is shown in the ‘After randomization’

The primary objective of this clinical study is to evaluate the efficacy and safety of bone regeneration therapy using tissue engineering materials including BM-MSCs in patients with maxillomandibular bone defects resulting from maxillofacial injury, or tumor/cyst removal. The TEOM study is an open-label, single-center (Nagoya University Hospital, Japan), randomized controlled study involving a total of 83 regions (BM-MSCs group: control group = 55 regions: 28 regions) by the Fédération Dentaire Internationale numbering system in patients with large jawbone defects. Maximum patient number is 29 (BM-MSCs group: control group = 19 patients: 10 patients) with at least 3 defect segments per patient thereof. A flow diagram of the TEOM study is shown in Fig. 1. The primary endpoint of this study is the rate of procedure sites with successful bone regeneration that satisfies both of the following definitions; i) CT value is at least 400, and ii) Bone height is over 10 mm. We decided as a specific hypothesis that the rate of patients who fulfil the primary endpoint criteria is similar in both groups, and the non-inferiority margin is 15.0%. Additionally, we will consider whether or not secondary endpoints in the BM-MSCs group will be superior to those in the control group.

### Selection of subjects

In this study, we would like to examine the effect of BM-MSCs in patients with larger bone defects than those in our previous reports [10, 13–19]. Therefore, we decided to select patients who have a maxillomandibular bone defect that comprises over 1/3 of the maxillomandibular area, because implant surgery after bone regeneration in this defect size is reimbursed under Japanese National Health Insurance.

The detailed inclusion criteria are as follows:

1. Patients who require bone regeneration satisfying all of the inclusion criteria as follows: Patients who have a maxillomandibular bone defect that comprises over 1/3 of the maxillomandibular area with a remaining bone height of ≤10 mm due to maxillofacial injury or tumor/cyst removal. Patients who had surgical tumor removal in the maxillomandibular region must not have had a relapse/metastasis during a 5-year observation period following the procedure
2. Aged 20 years or above
3. Patients who have received plaque control instructions from a dentist or a dental hygienist and are in good management of it
4. Patients that can provide signed informed consent

The main exclusion criteria are as follows:

1. Patients with bone defects as a result of periodontitis
2. Patients for whom surgical procedure is prohibited e.g. severe heart disease, congenital coagulation factor deficiency, leukemia, dialysis, end-stage malignant tumor
3. Patients with or with a history of infection e.g. Hepatitis B, Hepatitis C, HIV, HTLV
4. Patients with diabetes that is not well-controlled
5. Patients taking bisphosphonates for more than 3 months or denosumab for more than 6 months
6. Patients with or with a history of malignant tumor within the last 5 years
7. Patients taking steroids or immunosuppressive drugs who cannot stop such medication for 4 weeks after trial intervention
8. Patients in a serious condition of certain diseases (e.g. collagen disease, metabolic bone disease, immunodeficiency disease, metabolic disease, endocrine disease, blood disease, liver disease)
9. Patients with difficulty in bone marrow aspiration
10. Patients with difficulty in achievement of Hb ≥ 11 g/dL at preoperative examination
11. Patients with contraindications to local anesthetics
12. Patients with a history of hypersensitivity to antibiotics, antifungals, thrombine products, or bovine blood-derived products
13. Patients taking hemocoagulase products, tranexamic acid products, or aprotinin products
14. Female patients who are pregnant, suspected to be pregnant, breastfeeding, or those who do not agree to practice contraception
15. Any other patients whom the trial investigator deems ineligible for this study

### Registration and informed consent

Patients are registered as candidates for this study after providing a written informed consent form. After the investigators have confirmed that they meet all eligibility criteria, the patients are enrolled via an electronic data capture system (Viedoc™, PCG Solutions Ab. Uppsala, Sweden), with subsequent randomized assignment to either the BM-MSCs group or control group by the minimization method. Allocation factors are shown as follows: i) the number of regeneration segments (< 4 segments, 5 to 6 segments, > 7 segments), ii) the method of applied operation (sinus floor elevation, any other method of operation, sinus floor elevation with any other method of operation), bone regeneration site (maxilla, mandibular, both).

### Preparation and application of tissue engineered materials

Autologous BM-MSCs are cultivated from the iliac crest bone marrow aspirate for about 6 to 8 weeks as per previously reported methods [10, 13–19]. Before incubation of BM-MSCs, the adequate quantity of serum is prepared by dividing the blood samples several times. Moreover, the patient’s own PRP is prepared on the day before the application of tissue engineered materials. The other components of the tissue engineered materials, thrombin (Thrombin oral/topical 5000 units “JB” or Thrombin oral fine gran. 5000 units), calcium (Calcium Chloride “Yamazen”), and β-TCP (OSferion) are purchased from Japan Blood Products Organization (Tokyo, Japan), Yamazen Corporation (Osaka, Japan), and Olympus Corporation (Tokyo, Japan), respectively.

**Fig. 2:**
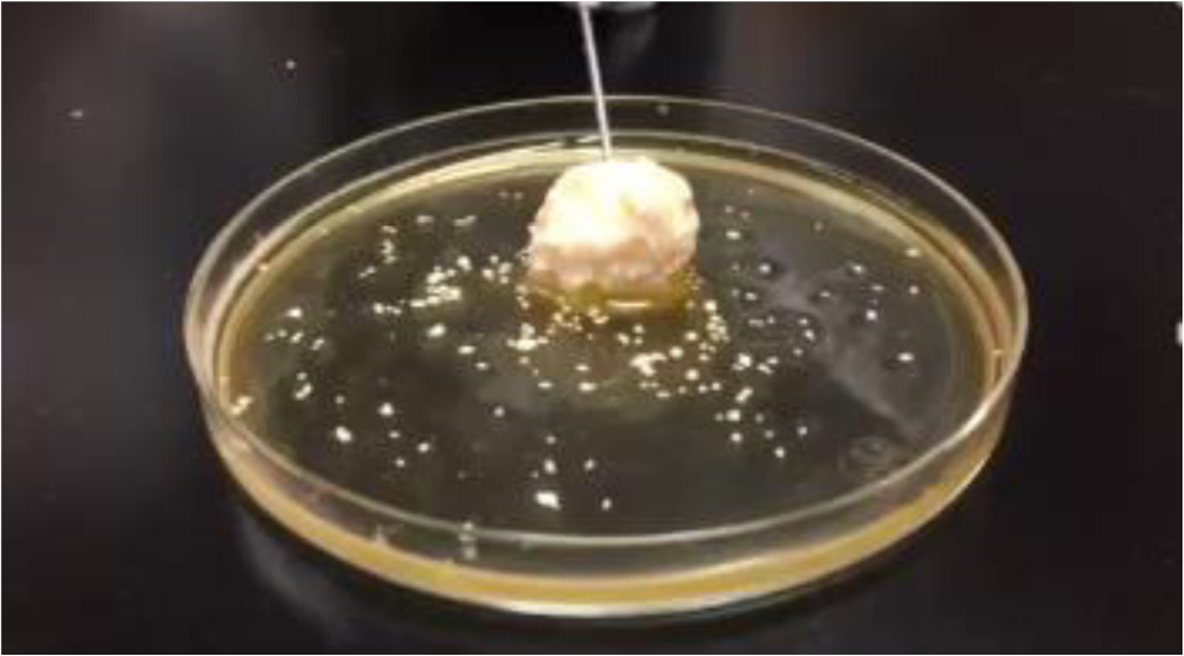
The appearance of the mixture of BM-MSCs, PRP, thrombin, calcium, and β-TCP. The mixture of BM-MSCs and PRP is combined with the thrombin-calcium solution, and then these contents start to have a gel-like appearance. After that, they are mixed with β-TCP

A 10% Calcium chloride solution is prepared in advance, and thrombin (5000 units) is dissolved in 5 mL of the 10% calcium chloride solution. The mixture of BM-MSCs (about 1 × 10^7^ cells) and PRP (2.0 mL per 3.0 mL defect) is combined with the thrombin solution (0.5 mL per 3.0 mL defect). After the contents start to have a gel-like appearance, they are mixed with β-TCP (0.5 g per 3.0 mL defect). The appearance of the mixture of BM-MSCs, PRP, thrombin, calcium, and β-TCP is shown in Fig. 2. BM-MSCs are not included in this mixture for the control group. Next, the mixture is injected to the bone augmentation site following applied operation such as guided bone regeneration, or sinus floor elevation after meticulous debridement.

Incidentally, Bisphosphonate and denosumabu are prohibited during the trial in order to modify bone metabolism and have the risk of osteonecrosis of the jaw.

### Response variables (outcomes)

Patients will be followed-up for 24 months after transplantation of materials. The TEOM study schedule is shown in Table 1. All data are collected via an electronic data capture system (Viedoc™, PCG Solutions Ab. Uppsala, Sweden), and checked by data manager and monitor according to the data management and monitoring plan.

**Table 1:** The TEOM study data collection schedule

	Screening	Preparation for operation				Operation	Observation period					
	Registration and assignment	† Blood sampling	† Bone marrow sampling	Preoperative examination	Day − 1	Day 0	Day 1	Month 1	Month 3	Month 6	Month 12	Month 24
Eligibility criteria	X											
Vital signs^‡^		X		X		X	X	X				
Laboratory tests												
Infections^§^, HbA1c	X											
Hematology^¶^, Biochemistry^††^, CRP, Coagulation^‡‡^	X	X^§§^		X			X	X	X	X	X	X
Urinalysis^¶¶^				X			X	X	X	X	X	X
Blood sampling		X			X							
Bone marrow sampling			X									
Panoramic X-ray	X						X	X	X	X	X	X
Facial CT	X						X	X	X	X	X	X
Tooth mobility^†††^	X								X	X	X	X
Masticatory force	X							X	X	X	X	X
Biopsy of bone (if needed) for histology								X	X	X	X	X
Transplantation of tissue engineered materials						X						
Concomitant therapies	X	X	X	X	X	X	X	X	X	X	X	X
Adverse events		X	X	X	X	X	X	X	X	X	X	X

† Only BM-MSCs group

‡ Blood pressure, pulse rate, body temperature

§ HBs antigen, HCV antibody, HIV antibody, HTLV-1 antibody, serologic test for syphilis, PVB19 antibody

¶ Red blood cell count, hemoglobin, hematocrit, white blood cell count, fraction of leucocytes (basophil, eosinophil, neutrophil, lymphocyte, monocyte), platelet count

†† Total protein, albumin, total cholesterol, blood urea nitrogen, creatinine, uric acid, sodium, chloride, potassium, calcium, phosphate, lactate dehydrogenase, aspartate aminotransferase, alanine aminotransferase, alkaline phosphatase, gamma-glutamyl transferase, total bilirubin

‡‡ Prothrombin time, activated partial thromboplastin time, fibrinogen

§§ Only red blood cell count, hemoglobin, white blood cell count, platelet count

¶¶ pH, protein, glucose, urobilinogen, occult blood

††† After setting of dental implant

Abbreviations: CRP, C-reactive protein; CT, Computed Tomography

As a general rule, the primary endpoint should reflect clinically relevant and meaningful effects [21]; therefore, we selected the rate of implanted sites with successful bone augmentation as the primary endpoint of the TEOM study. The definition of successful bone regeneration is the consideration as to whether the strength of augmented bone site is able to place a dental implant or not.

The secondary endpoints are as follows:

1. Regenerated bone height by facial CT and panoramic X-ray image
2. Percentage of the regenerated bone height over the height of the initial defect by facial CT and panoramic X-ray image
3. CT value
4. Rate of successful implant placement
5. Time from transplantation to implant placement
6. Survival rate and time of dental implant
7. Tooth mobility by the implant stability meters from Osstell (SASAKI Co., Ltd. Aich, Japan)
8. Masticatory force by Occlusal Force-Meter (GM10, Nagano Keiki. Tokyo, Japan)
9. Histological assessment
10. Adverse event
11. Laboratory tests

In addition to achievement of the primary endpoint criteria, the clinical meaning of additional BM-MSCs in bone augmentation will be considered comprehensively according to these secondary endpoint results.

### Data and safety monitoring committee

An independent data and safety monitoring committee is established in this study for assessment of the safety data in order to decide whether to continue, modify, or stop a trial, and performed according to the standard operating procedure.

### Sample size calculation

We assume that the rates of ‘Successful bone regeneration’ as a primary endpoint are commonly 95% in the two groups based on our clinical pilot study [14]. The non-inferiority margin is set to be 15.0% as a clinically acceptable difference.

With 83 sites per group, there was 80% power to examine the non-inferiority of the BM-MSCs group relative to the control group under the assumption of a two-sided significance level of 5.0% and an allocation ratio of 2: 1 to the BM-MSCs group or control group.

### Statistical analysis

All the analysis is based on an intention to treat principle. The primary analysis is to estimate the differences in the rate of ‘Successful bone regeneration’ and its 95% confidence interval (CI) based on the Clopper-Pearson method. The non-inferiority of the BM-MSCs group relative to the control group is evaluated based on whether the lower limit of the 95% CI exceeds the non-inferiority margin of 15%. The descriptive statistics for continuous variables and frequency and proportion for categorical variables are calculated. The time-to-event data are summarized by using the Kaplan-Meier method. All the statistical tests use a two-sided *p*-value. *P* < 0.05 is considered to be statistically significant. All the statistical analyses are performed by using the SAS version 9.4 (SAS Institute, Inc., Cary, NC, USA), and performed according to the statistical analysis plan.

### Audit

A systematic and independent examination were conducted according to the protocol, standard operating procedures, and the applicable regulatory requirements.

## Discussion

Recently, there have been many reports of cell therapy in tissue engineering approaches, although, these evaluations have not been established clearly. Our basic research revealed that cultured BM-MSCs improved the volume and hardness of regenerative bone in a dog model of bone defects [10–12]. Although these promising basic research results drove us to implement clinical studies, our clinical studies were case series or case reports in patients with relatively small atrophy or defects [10, 13–19]. In a systematic review of a regenerative approach to edentulous maxilla [22], it was concluded that ‘clinical trials assessing meaningful outcomes, involving larger populations, and with longer follow-up are warranted to discern the effectiveness of the achieved results compared with a valid control.’ In this review, two reports are cited as randomized controlled trials in patients with severe vertical or combined defects, but these clinical study designs both were the split-mouth design in the comparison effect between PRP in combination with autologous bone and autologous bone alone [23, 24]. Accordingly, there are no randomized controlled trials of cell therapy in severe atrophy or defects in the jawbone as far as we know. Therefore, we intended to implement this trial as the randomized control trial for evaluating the efficacy and safety of BM-MSCs in patients with large maxillomandibular bone defects.

As above, we constructed the rationale of the main design as a randomized controlled clinical trial for maxillomandibular bone defects based on our previous research [10–19]. Then, we discussed the design of the investigator-initiated clinical study (the TEOM study) in a consultation meeting with MHLW. As a result of a series of discussions, the Advanced Medical Treatment Council of the MHLW accepted our proposal.

### Ethics approval and current status on this trial

This study protocol is in accordance with the Declaration of Helsinki [25], the ‘Act on the Safety of Regenerative Medicine’ in Japan, and notifications of the Advanced Medical Care program from MHLW. Personal information management is in accordance with the protection regulation in Nagoya University. The compensation and/or treatment is available to the subject in case of the trial-related adverse event in accordance with the compensation regulation in Nagoya University.

The protocol of this trial was approved by the Certified Special Committee for Regenerative Medicine of The Japanese Association for the Promotion of State-of-the-Art in Medicine in November 2015 (No. PB4150004), however in January 2017 we changed the reviewing ethics committee to Nagoya University. Moreover, in December 2015, a clinical trial notification was accepted by the Advanced Medical Treatment Council of the MHLW (No. 1218–3). Protocol modification is also reviewed by these committees. The first patient completed registration in May 2016 and received transplantation of tissue engineered materials in July 2016. The TEOM study has currently enrolled 28 segments in 4 patients and is still recruiting patients from the Nagoya University Hospital. We are planning to enroll 83 regions as the full analysis set. The limit of enrollment will be January 2020, and the planned study end is July 2022.

## Conclusion

We present herein the overall design of this open-label, single-center, randomized controlled study to evaluate the efficacy and safety of tissue engineered osteogenic materials in bone defect patients. In this article, we provide the key considerations regarding the overall study design, selection of subjects, registration, application of tissue engineered materials, response variables (endpoints), number of subjects and statistical analysis. When this trial is completed or prematurely terminated, the clinical study reports are prepared according to the standard operating procedure, and submitted in peer review journal.

The TEOM study is the first randomized controlled clinical trial of a regenerative treatment for patients with maxillomandibular bone defects using BM-MSCs derived using our cultivation methods. We will evaluate efficacy and safety in this trial to provide an exploratory basis for the clinical meaning of additional cells in bone augmentation.

## Abbreviations

BM-MSCs: Bone marrow-derived mesenchymal cells
CI: Confidence interval
CT: Computed tomography
MHLW: Ministry of Health, Labour and Welfare
PRP: Platelet-rich plasma; QOL: quality of life
β-TCP: Beta-tricalcium phosphate
